# OVarFlow: a resource optimized GATK 4 based Open source Variant calling workFlow

**DOI:** 10.1186/s12859-021-04317-y

**Published:** 2021-08-13

**Authors:** Jochen Bathke, Gesine Lühken

**Affiliations:** Institute of Animal Breeding and Genetics, Justus Liebig University Gießen, Ludwigstraße 21, 35390 Gießen, Germany

**Keywords:** Variant calling, SNP, indel, GATK, Next generation sequencing, Reproducibility, Data parallelization, Benchmarking, Java

## Abstract

**Background:**

The advent of next generation sequencing has opened new avenues for basic and applied research. One application is the discovery of sequence variants causative of a phenotypic trait or a disease pathology. The computational task of detecting and annotating sequence differences of a target dataset between a reference genome is known as "variant calling". Typically, this task is computationally involved, often combining a complex chain of linked software tools. A major player in this field is the Genome Analysis Toolkit (GATK). The "GATK Best Practices" is a commonly referred recipe for variant calling. However, current computational recommendations on variant calling predominantly focus on human sequencing data and ignore ever-changing demands of high-throughput sequencing developments. Furthermore, frequent updates to such recommendations are counterintuitive to the goal of offering a standard workflow and hamper reproducibility over time.

**Results:**

A workflow for automated detection of single nucleotide polymorphisms and insertion-deletions offers a wide range of applications in sequence annotation of model and non-model organisms. The introduced workflow builds on the GATK Best Practices, while enabling reproducibility over time and offering an open, generalized computational architecture. The workflow achieves parallelized data evaluation and maximizes performance of individual computational tasks. Optimized Java garbage collection and heap size settings for the GATK applications SortSam, MarkDuplicates, HaplotypeCaller, and GatherVcfs effectively cut the overall analysis time in half.

**Conclusions:**

The demand for variant calling, efficient computational processing, and standardized workflows is growing. The Open source Variant calling workFlow (OVarFlow) offers automation and reproducibility for a computationally optimized variant calling task. By reducing usage of computational resources, the workflow removes prior existing entry barriers to the variant calling field and enables standardized variant calling.

## Background

Evolution, and thus the diversity of life, is based on genetic variability. This may arise from small changes in the nucleotide sequence of an organism, from larger rearrangements, from recombination of homologous chromosomes during chromosomal crossover, or from chromosome reshuffling during meiosis and sexual reproduction [1–3]. As a result, new phenotypic traits may be realized within an individual. Identifying the genetic basis of such phenotypic traits is a pivotal point of genetic studies. In particular, the advent of next generation sequencing (NGS) technologies has ushered in a new era of sequencing based genetic variant identification [4, 5]. Continuous improvements in second and third generation sequencing technologies promoted permanently declining sequencing costs and even surpassed the technological advances in the semiconductor industry described by Moore’s law [6]. This paved the way for new applications of NGS methods and a broader application thereof. In this regard, NGS even challenges SNP genotyping arrays for various genome analysis applications [7]. Whole genome (WGS) as well as whole exome sequencing (WES) are now commonly used for variant discovery, with a trend towards WGS [8], even though WES is still relevant [9]. Genome-wide association studies (GWAS), previously the domain of SNP genotyping arrays [10], are increasingly conducted using WGS [11, 12].

Generating the sequencing data is only the first step in any related research project. Major steps in subsequent analyses include read mapping, variant calling, variant filtration and functional annotation of the variants [13, 14]. Over the last decade a plethora of variant callers have been developed [15]. The Genome Analysis Toolkit (GATK) is among the most widely used applications [16] and GATK Best Practices workflows are considered a kind of gold standard in the field [17–19]. The GATK includes hundreds of different tools and the GATK Best Practices are intended to guide users through their application [13, 17]. Therefore, it has become customary to simply cite the GATK Best Practices in method sections of publications, while supplying a link to the GATK website [20–22]. The problems resulting from this routine are twofold. Firstly, the GATK Best Practices are a dynamic document in which command lines, arguments, and tool choices can become obsolete. Years after the initial publication, it might become elusive what the GATK Best Practices were by that time. Secondly, simply citing the GATK Best Practices is used as a shortcut to write method sections. This has also been noticed by the developers of the GATK, therefore stating on their website: “Lots of workflows that people call GATK Best Practices diverge significantly from our recommendations [23].” Reproducibility of the actual data evaluation is thus obscured, which relates to the fact that the “Best Practices workflows […] are designed specifically for human genome research [23]” even though the GATK has successfully been used to analyze various species [20, 22, 24, 25].

Much scientific work is unnecessarily protracted by the lack of reproducibility due to unclear methods [26]. Reproducibility is as much a concern in computational biology as it is in laboratory work [27]. In particular, complex workflows such as variant calling and software stacks such as the GATK constitute a challenge to small scientific groups and newcomers to the field.

The rapidly growing adaptation of NGS-based variant calling, the widespread use of the GATK, and the need for reproducible data analysis highlight a demand for broadly applicable, well documented, and readily usable GATK-based variant calling workflows. We therefore developed OVarFlow, an open source, highly optimized, full-fledged variant calling workflow that generates functionally annotated variants. The workflow is highly automated and reproducible, requiring minimal user interaction. Only short read sequencing data (e.g. Illumina), a reference genome, as well as annotation need to be provided.

## Implementation

The analysis of sequencing data is highly dependent on software tools, where usability, installability, and archival stability are key aspects for the usefulness of software tools [28]. A systematic analysis has shown that a large proportion of published tools cannot be readily installed due to implementation issues [28].

To circumvent such nuisance, OVarFlow comes with a comprehensive documentation and builds upon established technologies. These include Conda [29] and Bioconda [30] as an environment and package manager, allowing for easy installation of the required software components. Snakemake is utilized as a workflow management engine to orchestrate the various data processing steps [31]. Alternatively, all required software components come bundled via container virtualization as Docker [32] or Singularity [33] containers, if manual intervention during setup is not desirable. Data processing itself is primarily relying on the GATK [16].

The complexity of computational methods is ever-growing. We are therefore convinced that a thorough documentation is essential for the usability of any software. For this reason, comprehensive documentation is an integral part of OVarFlow. It is available at “Read the Docs”, explaining in detail the setup, usage, resource requirements, and the individual steps of the workflow.

### Overview of the workflow

**Fig. 1:**
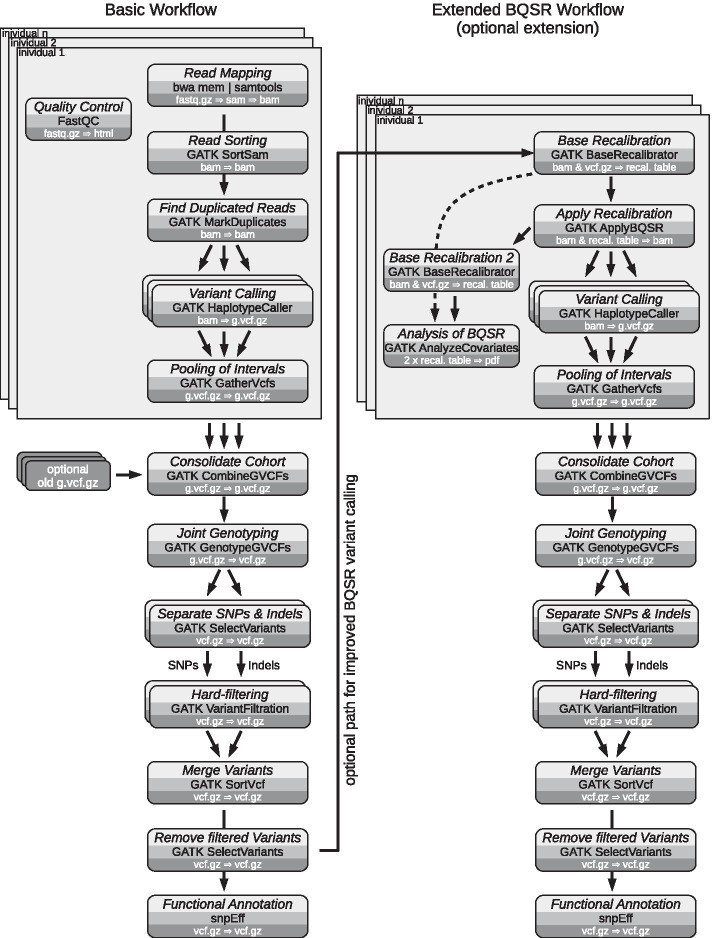
Flowchart of the variant calling workflow. The variant calling workflow consists of two separate branches. A basic workflow already generates a set of usable, functionally annotated variants (SNPs and indels). A second, optional workflow, uses the previously called variants to perform Base Quality Score Recalibration (BQSR) to improve initial base calls of the fastq files. Processing of each individuals fastq files can be performed in parallel. Also various steps of the workflow can be parallelized, e.g. base calling on genomic intervals by the GATK HaplotypeCaller, as indicated by overlapping boxes. Each box includes a description of the step (light gray), the name of the used application (medium gray) and the primary input and output data formats (dark gray)

A flowchart of the actual variant calling workflow is depicted in Fig. 1. The entire workflow consists of two separate branches. The basic variant calling workflow can already stand on its own, while an optional second, extended workflow allows for further refinement of the called variants via base quality score recalibration (BQSR), if desired.

The basic workflow requires a reference genome in fasta format (.fa.gz, optionally compressed) and reference annotation in general feature format (.gff.gz, also compressed). Finally, second generation sequencing (SGS) data have to be provided in fastq format (.fastq.gz). By providing these data, a complete set of functionally annotated variants is generated. Each step of the analysis is depicted in a rounded box, naming the analysis performed, the application used, and the primary input and output data types. Sequencing data of several individuals can be processed in parallel and are then consolidated into a single cohort, resulting in a genomics variant call format (VCF) file (g.vcf.gz). Hard-filtering is then employed to minimize the number of false positive variants. Finally, SnpEff performs a functional annotation of the called variants [34].

A second workflow can be executed in succession to the first one. Thereby the called variants can be further refined by BQSR, as initially shown by DePristo et al*.* [17]. Basically, BQSR tries to improve the quality scores generated by the sequencer. However, depending on the given data set, the improvements obtained by BQSR may be marginal while incurring high computational cost [35]. Therefore, BQSR has been included as an optional step that might be applied to low-coverage data or poorly calibrated base quality [35].

### Optimized parallelization of the workflow

Variant calling is a computationally demanding task. To speed up the analysis, parallelization has been enabled wherever possible. Firstly, fastq files of various individuals can be processed in parallel, up to the point where variants are consolidated into a single genomics VCF file (.g.vcf.gz). Also filtering of SNPs and insertion-deletions (indels) is performed in parallel. The two most demanding steps of the workflow are mapping via the maximal exact matches (mem) algorithm of the Burrows–Wheeler aligner (bwa) [36] and variant calling via the GATK HaplotypeCaller. Mapping via bwa can easily be parallelized and uses six threads in OVarFlow by default (configurable). The HaplotypeCaller, on the other hand, used to be parallelizable by means of command line switches (-nct and -nc), but these options where abandoned with GATK 4. As an alternative, the GATK team introduced Spark for multithreading [37]. At the time of writing, HaplotypeCallerSpark (version 4.2.0 and below) “is still under development and should not be used for production work”, as stated by the developers [38]. OVarFlow reintroduces parallelization to the HaplotypeCaller by splitting the reference genome into several intervals on which variant calling is performed in parallel. This approach is also called “scatter gather”. The amount of intervals processed in parallel is configurable, but defaults to four. A python script that is part of the workflow, splits the reference genome between individual fasta sequences (contigs or scaffolds) into intervals. Individual sequences are not split. Furthermore, we examined the resource consumption of the HaplotypeCaller depending on the particular setting for its native pair hidden Markov model (HMM) implementation (command-line option–native-pair-hmm-threads; see results). While four native pairHMM threads gave the best runtime for the individual HaplotypeCaller process, more processes could be executed in parallel when only a single native pairHMM thread was enabled. This was made configurable in the workflow, while defaulting to four native pairHMM threads. Finally, the entire analysis scales with the given hardware. Here, Snakemake helps to achieve optimized resource utilization and parallelization from high-end desktops to server to cluster usage [31].

### Pitfalls when working with the GATK

The GATK is a very complex framework with hundreds of applications related to variant calling. Many of those show some very intricate and subtle usability issues that are challenging for both new and expert users. One of those pitfalls involves hard filtering, which is often employed to remove false positive variants [13, 39]. Often, hard filtering is applied by concatenating several filtering expressions using the logical or-operator “||” [25]. This may be due to a 2013 publication by the authors of GATK in which this was suggested [13]. This approach has changed. As of today, the GATK team states, “it is strongly recommended that such expressions be provided as individual arguments … [to] ensures that all of the individual expression are applied to each variant” [40]. Therefore, OVarFlow applies a separate filter to each filtration threshold (for SNPs: QD < 2.0, QUAL < 30.0, SOR > 3.0, FS > 60.0, MQ < 40.0, MQRankSum < -12.5, ReadPosRankSum < -8.0; for INDELs: QD < 2.0, QUAL < 30.0, FS > 200.0, ReadPosRankSum < -20.0).

Another potential pitfall is the CPU instruction set. Performance of the GATK 4 has been optimized in an collaboration between Intel and the Broad Institute. This includes the use of advanced vector extensions (AVX) for the HaplotypeCaller [41, 42]. The absence of AVX results in a drastic slowdown of the HaplotypeCaller (personal estimates are about 5 times longer runtimes). OVarFlow therefore verifies the availability of AVX before executing the workflow and informs the user about the absence of AVX. Fortunately, AVX evolved to be common in newer CPU generations.

The GATK is written in the Java programming language, which is inherently linked to the Java virtual machine (JVM). Performance of the JVM can be optimized by several hundreds of settings (see: java -XX: + PrintFlagsFinal and java -X). In particular, the Java heap size and number of garbage collection threads exert a major influence on the performance of various GATK applications (see results section). In order to achieve an optimized resource utilization and runtime, these two parameters were optimized for the most important tools running in parallel and incorporated into the workflow.

Moreover, several minor inconveniences are handled by the workflow. These include the storage of intermediate data under the /tmp direcotry. Depending on the partition scheme and size, this can be a source of major trouble. Therefore, temporary data are stored under GATK_tmp_dir within the project directory. Depending on the input data, the GATK application MarkDuplicates opens a plethora of files, which is even more problematic since multiple instances of MarkDuplicates can run in parallel. To avoid problems arising from the maximum number of allowed open file descriptors (see ulimit -Sn or -Hn), each instance of MarkDuplicates has been limited to use 300 file handles. Finally, the workflow saves the user from fiddling around with many intermediate files, including various indices (.bai,.fai,.dict, bwa index), conversion of the gzip to BGZ format [43], or creation of a SnpEff database.

### The configuration files

The user is freed from as much manual intervention as possible, all while scaling across different infrastructure sizes. This streamlining of the data evaluation is also achieved by “convention over configuration”. However, configurability is implemented through two configuration files, one of which is entirely optional.

The first configuration file (samples_and_read_groups.csv) describes the input data. This file specifies the more “biological” data, such as the reference genome and annotation, as well as the sequenced samples. It is in colon separated values (csv) format, which allows for easy editing even in common spread sheet applications. Furthermore, read group data have to be specified in this file. Additionally, previously called variants can be included in the analysis. In addition, a minimum sequence length can be specified. Many genomes contain a high number of small contigs. These can be excluded from the analysis by setting the desired cut-off value.

A second, optional configuration file (config.yaml) deals with the more “technical” side of the workflow. If present, this file is automatically picked up by the Snakemake workflow management system. Here, the Java heap size and the number of garbage collection threads can be adjusted if required. Parallelization can also be configured for the number of bwa threads, intervals of the genome that can be processed in parallel by the HaplotypeCaller, and the number of native pairHMM threads used by the HaplotypeCaller. Finally, storage of temporary data and the maximum number of file handles used by MarkDuplicates can be configured. A separate configuration file is available for the BQSR workflow (configBQSR.yaml), which serves identical purposes.

## Results

The primary goal of our workflow is for it to be of practical use in variant calling projects. To demonstrate its validity, we reproduced the variant identification from a previous study. Another matter of concern was the resource efficiency of the variant identification. The potential to improve the performance of GATK based variant calling has been shown previously [42]. Here, we build upon those findings and further extend them into a lean variant calling workflow. For that reason, we investigated how to optimize resource utilization of individual GATK applications and also on the level of the entire workflow.

### Proof of concept

To confirm the feasibility of our workflow, we reproduced the identification of a variant responsible for recessive autosomal dwarfism (adw) in chicken, as originally performed by Wu et al. [44]. The adw variant was known to be located on chromosome 1, within 52–56 Mb. Variant identification was performed using WGS data from a single *adw/adw* individual and 261 unaffected White Leghorns as controls. A total of 146,070 variants could be identified within the candidate region, which were further reduced by various filtering steps to 11 potential candidates, of which only one was categorized as a high impact variant (stop gained).

To repeat this analysis, we obtained the sequencing data of the *adw/adw* individual from the European Nucleotide Archive (ENA, www.ebi.ac.uk/ena), along with raw read data of another 25 normal White Leghorns. The fastq sequencing data of these 26 chicken served as input data for the variant calling workflow presented here. To retain comparable genome coordinates, the same reference genome (Gallus_gallus-5.0) was utilized as in the previous study. The following steps were similar to the data evaluation procedure of Wu et al. Briefly, all variants within the candidate region of 52–56 Mb on chromosome 1 were extracted. All variants that were not homozygous and also not exclusive to the dwarf chicken were removed from the dataset. This resulted in a total of 1,090 variants. Those variants that were categorized by SnpEff as having moderate or high impact were finally selected, yielding a total of 6 potential candidate variants (see Table 1). Most of these variants posses multiple annotations and exceed different impacts on the respective annotation. The identified candidate variant set is not identical to the 11 candidates found by Wu et al., which is not surprising, giving the fact that different White Leghorns were used as a reference dataset as compared to the original study. Variants identified as potential candidates in both studies are marked with a small check mark, and those exclusive to our analysis are marked with a cross. However, all of those 11 variants selected by Wu et al*.* could be identified in our dataset prior to the filtering step (only the SNP at position 52,195,787 was detected as a deletion at position 52,195,786). More importantly, the causative variant (position 53,688,583) that was finally identified by Wu et al*.* was among the 6 candidates identified by our analysis.

**Table 1:** Variants exclusively associated with autosomal dwarfism in chicken

Position	Ref/Alt	Gene	Category	Impact	Wu et al
53,369,406	G/A	ASCL4	Missense variant Non coding transcript variant	Moderate Modifier	✔
53,406,379	C/G	PWP1	Missense variant Non coding transcript variant	Moderate Modifier	✘
53,688,583	C/T	MTERF2 C1H12ORF23	Stop gained Downstream gene variant Non coding transcript variant	High Modifier Modifier	✔ causative variant
54,232,753	G/A	C12ORF75	Missense variant	Moderate	✔
54,593,287	G/A	CHST11	Missense variant	Moderate	✘
54,764,182	CT/C	0	Frameshift variant Non coding transcript variant	High Modifier	✘

Taken together, these results confirm that our variant calling workflow is capable of detecting small variants (SNPs and indels) in whole genome sequencing data that can be utilized in further analysis steps to identify potential causative variants. In addition to chicken, we also tested OVarFlow with a variety of different organisms and various assemblies obtained from the RefSeq, including chicken, duck, *C. elegans*, sheep, pig and alpaca. In particular, the alpaca genome (GCF_000164845.2) was highly fragmented (276,725 scaffolds), but variants were still successfully called.

### Optimization of individual GATK applications

The GATK is written in the Java programming language, whose bytecode is executed by the JVM. One issue with the JVM is that its resource utilization does not always scale positively with the size of given hardware resources. This is particularly problematic, as variant calling is resource hungry and requires large computational resources.

Two aspects of the JVM are automatically adjusted to the available hardware, namely the number of garbage collection (GC) threads and the heap size (see Table 2). Both of these aspects relate to memory management by the JVM (here version 8). They were measured on actually available hardware. While the number of GC threads grows continuously with the number of given CPU cores, the heap size maxes out at 26.67 Gb. In many cases, these numbers are considerably larger than what is needed for the respective application to run efficiently, as shown by the following measurements. This also means that GATK applications will exhibit inconsistent behavior depending on the given hardware.

**Table 2:** Default resource usage of the Java virtual machine version 8

Given CPU cores	Default GC thread count	Given system memory (Gb)	Default maximum heap size (Gb)
8	8	16	3.48
28	20	64	13.98
64	43	256	26.67
160	103	512	26.67
		1024	26.67

**Fig. 2:**
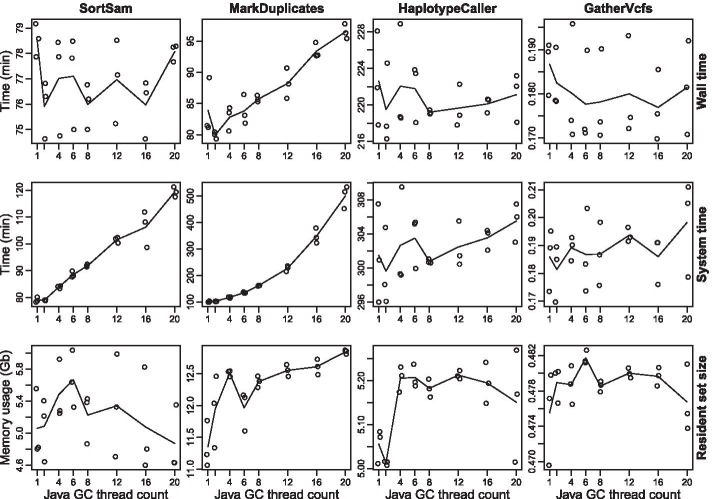
Resource usage benchmarking of GATK applications at different Java garbage collection thread counts. The performance of some GATK applications is severely influenced by the number of employed Java garbage collection (GC) threads. Each application was executed several times with different Java GC thread counts, intending to identify GC thread counts that result in minimal resource utilization. Here, the Java 8 default parallel garbage collector was used. Resource usage concerning wall time, system time and resident set size (memory usage) was analyzed (see rows) for the four tools SortSam, MarkDuplicates, HaplotypeCaller and GatherVcfs (see columns) (GATK version 4.1.9). Triplicated measurements for each of eight different numbers of GC thread counts (1, 2, 4, 6, 8, 12, 16 and 20) were recorded and resulting mean values plotted in lines. Lower measured values are preferable as they reflect a lower resource usage of the respective application. Runtime comparisons between different applications should not be performed here. The ordinate scales of individual plots vary greatly, to represent variances within an application as clearly as possible. Furthermore, SortSam, MarkDuplicates and GatherVcfs analyzed an entire dataset, while the HaplotypeCaller was limited to the analysis of chromosome 6 (NC_006093.5), thereby reducing the runtime from days to some hours

First, we assessed the impact of GC thread count on several GATK applications (Fig. 2). We selected those applications for a deeper analysis that are run in parallel. This part of the workflow generates the highest load and will benefit the most from optimization. Three aspects of each application where analyzed, namely overall execution speed (wall time), total load caused (system time), and memory usage (resident set size; RSS). Whenever possible, it is desirable to minimize all of these parameters, as this results in lower overall resource utilization. Wall time of GATK SortSam, HaplotypeCaller and GatherVcfs is barely influenced by the number of GC threads. MarkDuplicates shows a linear relation, resulting in longer runtimes at higher thread counts and a sweet spot at two GC threads. Total CPU usage for SortSam and MarkDuplicates increases with higher GC thread numbers. SortSam causes about 50% more CPU utilization between low thread counts and 20 GC threads. For MarkDuplicates the effect is even more pronounced, resulting in an approx. fivefold higher resource utilization between 1 and 20 GC threads. For HaplotypeCaller and GatherVcfs, no equally clear tendency is seen. Besides statistic variation, differences in memory consumption are not as pronounced. The GATK HaplotypeCaller might benefit from two GC threads.

From those findings, it can be concluded that memory footprint is only marginally influenced by the number of GC threads. CPU load, on the other side, is significantly affected for SortSam and MarkDuplicates, with the sweet spot being two GC threads for each.

**Fig. 3:**
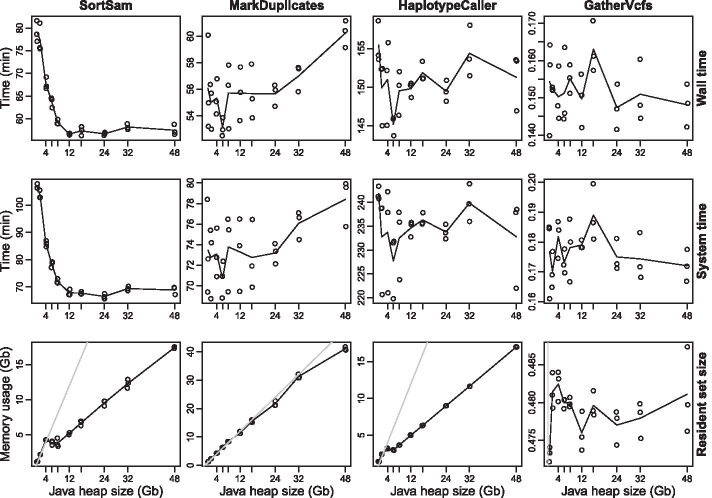
Influence of different Java heap sizes on the resource utilization of individual GATK applications. Besides the number of Java garbage collection threads, the provided heap size has a considerable impact on the performance of some GATK applications. Again, the four tools SortSam, MarkDuplicates, HaplotypeCaller and GatherVcfs (see columns) (GATK version 4.1.9) were assessed for their respective resource usage in terms of wall time, system time and memory usage (see rows). The intention was to identify Java heap sizes that result in minimized resource utilization. Therefore, lower readings on the ordinate are preferable as they reflect lower resource consumption of the respective application. Triplicate measurements were recorded for each of ten different values for Java heap size (1, 2, 4, 6, 8, 12, 16, 24, 32 and 48 Gb) and resulting mean values plotted in lines. The gray line in the resident set size plots indicate parity between the maximum allowed heap size and the actual memory usage. All measurements were recorded with two garbage collection threads enabled. As in Fig. 2, different scales of the ordinates of each plot have to be taken into account, since they vary considerably between the individual plots. In additon, the HaplotypeCaller was again limited to the analysis of chromosome 6 (NC_006093.5)

Next, we investigated the effects of different JVM heap sizes on the performance of the same applications, observing the same parameters as before (Fig. 3). The picture is clearly distinct. The impact on wall and system time are identical for each individual application. Especially, CPU usage of SortSam benefits from larger heap sizes. Here, 12 Gb is the sweet spot for the given data set. MarkDuplicates seems to benefit slightly from smaller heap sizes, but no definitive number can be given due to statistical fuzziness. Memory footprint is severely affected by the maximum allowed heap size for SortSam, MarkDuplicates, and HaplotypeCaller. In particular, MarkDuplicates scales linearly with the maximum allowed heap size and uses all the memory allocated as heap space (grey line). This is especially noteworthy since CPU usage and application runtime do not benefit from larger heap sizes. With SortSam being the exception, up to 12 Gb of heap space. Effects on GatherVcfs are negligible.

It can be concluded that the heap size exerts a drastic impact on memory consumption and, in case of SortSam, also on CPU utilization. To minimize memory usage of the workflow, SortSam was allowed to allocate up to 10 Gb heap space, while the other applications were limited to 2 Gb.

### Optimization of variant calling on the workflow level

Previously optimizations have been performed on the level of individual applications. In this section, we want to illustrate how the entire variant calling workflow can benefit from these optimizations. Furthermore, we are introducing additional optimizations on the level of parallelized data processing.

**Fig. 4:**
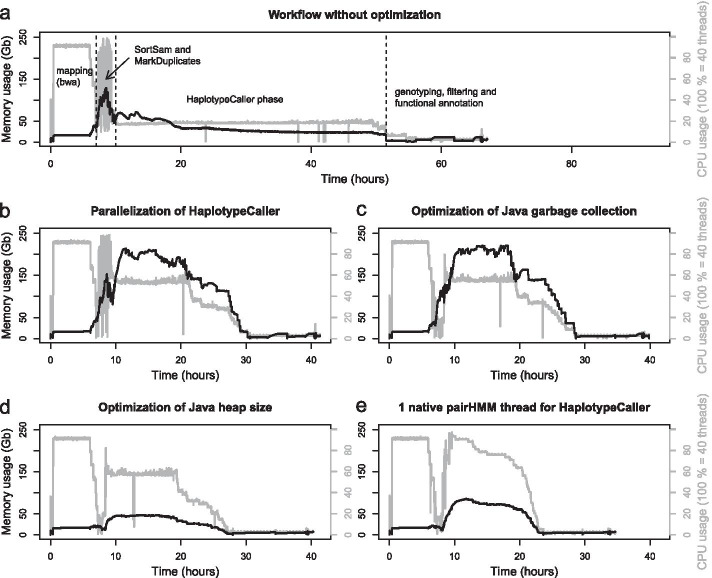
Resource consumption of the basic workflow with increasing optimization levels. **a** CPU and memory utilization of the entire workflow, using a single interval (comprising the entire genome) for the HaplotypeCaller and without any Java optimization (total runtime: 67.1 h)). Four phases can be distinguished within the workflow (separated by dashed lines), that are dominated by individual applications. **b** When the genome is split into six separate intervals for the HaplotypeCaller analysis, but without any Java optimization (41.4 h). **c** With optimized Java garbage collection for each GATK application (39.8 h). **d** With optimized Java settings (garbage collection and heap size) for all GATK applications and four default threads for the native pairHMM algorithm of the HaplotypeCaller (40.3 h). **e** When all optimizations are applied to the workflow, including six parallel intervals for variant calling by the HaplotypeCaller, a single hmmThread for each HaplotypeCaller, and all Java optimizations (garbage collection and heap size) (34.7 h)

CPU usage is only one aspect of hardware utilization. Memory usage can be another limiting factor, and shortage thereof can even result in out of memory related application termination. Therefore, both of these aspects were monitored during the entire runtime of the workflow (Fig. 4). Six fastq files with chicken whole genome re-sequencing data were used for these measurements. We chose chicken as it’s a vertebrate with a moderately sized genome of approximately 1.1 billion bases. This shortens the time for workflow benchmarking, while still allowing to estimate evaluation times for larger genomes.

Benchmarking of the workflow was performed for various degrees of GATK optimization. First (Fig. 4a), a baseline measurement was recorded without any particular GATK performance optimization. Parallelization solely relied on Snakemake’s scheduling of various processes to be executed simultaneously, and by bwa mem to use six threads for each distinct mapping process. The plateau in CPU utilization (gray line) within approx. the first six hours is due to mapping by bwa mem. After this phase, the workflow relies primarily on the GATK. Above all, the activity phases of SortSam, MarkDuplicates and the HaplotypeCaller were optimized.

Firstly, parallelization was applied to the HaplotypeCaller phase (Fig. 4b). This was achieved by splitting the reference genome into six discrete intervals, thereby artificially shortening the reference genome and allowing for more HaplotypeCaller processes to be executed in parallel. In doing so, CPU and memory usage are rising during the HaplotypeCaller phase (approx. between 10 and 30 h), while the overall runtime is shortened by more than an entire day. From this point on, resource utilization was further reduced through the previously optimized JVM settings.

Applying optimized GC thread values was able to reduce the first spike in memory utilization and caused an reduced and more even CPU usage between 6 and 10 h of workflow execution (Fig. 4c). This is mainly related to the more efficient resource utilization by SortSam and MarkDuplicates.

In the next step usage of the Java heap space was optimized (Fig. 4d). This allowed for a drastic reduction in memory utilization during the phase of parallel execution of SortSam, MarkDuplicates, HaplotypeCaller, and GatherVcfs (approx. 6 to 28 h). Before these optimizations were applied, memory utilization maxed out between 230 and 240 Gb and was lowered to a plateau at just about 50 Gb.

The resource utilization gains achieved above paved the way for additional optimization of the HaplotypeCaller phase (Fig. 4e). It was observed that the HaplotypeCaller exhibits an average CPU utilization of 140% at its default setting of four native pairHMM threads (for more data, see the documentation of OVarFlow, section “Benchmarking & Optimizations» Maximizing CPU utilization”), which means that one core is fully utilized and a second core is only 40% utilized. The Snakemake workflow management system, on the other hand, is only capable of calculating and scheduling entire threads. This means that it can be configured to schedule either a single CPU thread or two. Therefore, CPU usage of the HaplotypeCaller was assessed with different numbers of native pairHMM threads (see the documentation of OVarFlow). This showed that using one native pairHMM thread results in slightly longer runtimes, but also only a single thread is utilized. With this setting, the Snakemake scheduler only needed to allocate a single thread per HaplotypeCaller, effectively doubling the number of HaplotypeCaller processes executed in parallel. This maximized CPU utilization and reduced the runtime for the entire workflow by an additional 5 h.

Ultimately, all of these optimizations were incorporated into the variant calling workflow, but they were also made configurable. The settings can be adjusted via the configuration file config.yml or configBQSR.yml, respectively. This allows for a high degree of customization if needed, while reasonable defaults have been set within the workflow itself. Since the best performance for an individual HaplotypeCaller process is achieved at four native pairHMM threads, this was used as the default. This can lead to better runtime if unlimited hardware resource are available, e.g. in cluster usage. In this case Snakemake automatically schedules two threads per HaplotypeCaller process. Nevertheless, it can be configured to use just a single native pairHMM thread, in which case Snakemake reserves only a single thread.

## Discussion

The GATK is among the most popular variant calling frameworks [15, 45, 46]. Its Best Practices are commonly referenced as a data evaluation procedure, when writing method sections [17, 20]. However, it is often overlooked that the GATK Best Practices are very specifically tailored to human sequencing data [23]. This highlights a need for broadly applicable GATK based variant calling workflows. In addition, the Best Practices are constantly evolving, making it difficult to reproduce recommendations from several years ago. This is especially problematic for complex data evaluation procedures like variant calling, which involve more than a dozen computational steps and tools (Fig. 1), thereby compromising reproducibility. Poorly reproducible methods can also result in a loss of time, because a method or procedure must be partially reinvented [26]. We therefore created a variant calling workflow inspired by the GATK Best Practices for germline short variant discovery [47], but is more broadly applicable to both model and non-model diploid organisms.

The GATK is a very complex framework with hundreds of different tools, where the right tools for variant calling have to be identified first. Additional complexity is introduced by the JVM, which possesses hundreds of configuration options. A major pitfall is that the JVM does not always scale well with the size of the given hardware resources (see Table 2). The JVM tends to allocate more GC threads and larger heap size on a larger hardware base. This, in turn, can actually have a negative impact on the performance of several GATK applications. To many GC threads even slowed down SortSam and MarkDuplicates (Fig. 2), while unnessesary large heap spaces consumed more memory than necessary (Fig. 3). In the worst case, this can even result in out of memory errors, leading to program termination. The negative impact of too many GC threads for MarkDuplicates is consistent with previous findings, where various performance optimizations for the GATK were assessed [42]. In this study, the authors focused only on the execution time (wall time) of single applications. However, this is only one point to consider. System times are equally important, as they better reflect CPU utilization on multithreaded systems. This can be seen, for example, with SortSam (Fig. 2), where wall times remain identical regardless of the number of GC threads, but system times increased by approx. 50% between 1 and 20 GC threads, thereby diminishing the potential for parallel data evaluation. Memory consumption also contributes its share to the final bill. Optimization of heap usage reduced memory consumption at the workflow level to less than a quarter (Fig. 3c, d), without negatively impacting overall runtime. For one thing, this allows to achieve more with given hardware resources, for another thing, it might also save costs in cloud environments that consider memory usage in the bill. Here we not only show these performance optimizations, but have also implemented them in our final workflow, which has not been done in any general purpose GATK 4 based variant calling workflow that we are aware of. Besides our improvements to JVM settings, additional system level optimizations have already been assessed for multiple GATK 3.x versions [48]. Applying most of those optimizations requires administrator privileges, which is often not feasible in multi-user environments. Here, we focused on optimizations that can be applied by non-administrator users. Nevertheless, for large facilities, performing a lot of variant calling, this might be an opportunity to further accelerate variant calling.

Having a clearly defined data evaluation pipeline is only one aspect of scientific reproducibility. The most reproducible workflow is of no use if the underlying software cannot be installed. Mangul et al*.* conducted a systematic analysis of software installability, in which 51% of the investigated tools were claimed “easy to install”, yet 28% failed to be installed at all [28]. To circumvent installation obstacles and to promote the long-term usefulness of our workflow, we followed the general guidelines outlined by Manguel et al. Firstly, our software is hosted publicly by GitLab and the associated documentation is available via Read the Docs. Secondly, application installation relies on the well established Conda environment and package manager. Thirdly, Conda also takes care of all dependencies of the tools used. Furthermore, the last two points are also guaranteed by providing container images, which further reduces the effort on the end user's side. Additionally, the documentation includes not only an example dataset, but also a detailed tutorial on how to evaluate this dataset. Besides a quick start guide, a detailed description of every software component used is included. The software can be run without any root privileges. The Docker container might even be run on a Windows system. However, we strongly recommend performing variant calling on Linux based high-performance computing infrastructure.

Finally, our variant calling workflow is entirely based upon open source software. This is a key point for the unrestricted use and availability of our workflow. License changes between GATK 3 and 4 made this possible. With the previous license of GATK 3, distributing a prepackaged workflow in a container environment would not have been possible. This underlines the value of open source software and its associated licensing schemes, for the scientific community.

## Conclusion

Variant calling has become an established method in genomics research. Its utilization can only be expected to rise due to continuously declining sequencing costs. The GATK, developed at the Broad Institute, is a major player in this field and its Best Practices are commonly cited in related publications, despite being focused on human sequencing data. This demonstrates the high demand for an easily accessible and widely applicable GATK based variant calling workflow. The revised workflow fits this need, offering automatization, reproducibility, and documentation of the variant calling process. The presented workflow will help bridge the gap and further lower the threshold for variant calling. Taken together, the optimized workflow features transparent variant calling and economic computational resource management.

## Methods

### Utilized software versions

The following software versions were used within the variant calling workflow: FastQC v0.11.9, bwa 0.7.17-r1188 [36], samtools 1.11 [49], GATK 4.1.9 [16], and SnpEff 5.0 [34] (in order of use). Of these FastQC, GATK and SnpEff rely on the JVM for program execution. OpenJDK version 1.8.0_152 was utilized here. Snakemake [31] version 5.26.1 served as a workflow management engine. Software installation was performed via Conda 4.9.2. Default JVM resource usage was determined on various machines using the commands java -XX: + PrintFlagsFinal | grep ParallelGCThreads and java -XshowSettings:vm.

### Data analysis for dwarf chicken variant

Whole genome sequencing data were obtained from the ENA, for one dwarf chicken (ERR2505843) and 25 White Leghorns (ERR3525251-8, ERR4326765-74, SRR2131198-9, SRR2131201, SRR5079491-3, SRR5079496). The variant calling workflow was executed using chicken genome build Gallus_gallus-5.0. To reduce the total runtime, all contigs shorter than 20,000 bp were excluded by a mechanism build into OVarFlow. Variants on chromosome 1, within the candidate region 52–56 Mb, were extracted by the bcftools’ (1.6) view command. Filtering of potentially causal variants was performed using a custom Python 3 script, screening for homozygous variants exclusive to the dwarf chicken. Moderate and high impact variants, as categorized by SnpEff, were selected using the Unix command grep '\ (MODERATE\|HIGH\ )'.

### Benchmarking of individual GATK applications

To reduce application runtime, sequencing data from *Gallus gallus* were employed for performance benchmarking of individual GATK applications. The current representative genome GRCg6a (RefSeq assembly accession: GCF_000002315.6) was obtained from the RefSeq. Fastq files were obtained from the ENA, run accession SRR3041137, offering 2 × 125 bp Illumina sequencing data (HiSeq 2500). An average coverage of 34 with standard deviation of 44 was determined for this dataset (see rule calculate_average_coverage of the workflow for details). SortSam, MarkDuplicates, and GatherVcfs processed the entire dataset, while HaplotypeCaller was limited to chromosome NC_006093.5 (36,374,701 bp) to reduce application runtime. To benchmark various Java GC thread numbers the heap size was fixed to 12 Gb. Two GC threads were specified for the benchmarking of various Java heap sizes. Resource usage of each application was monitored by GNU time version 1.8 (version 1.7 reports incorrect results), written to a log file and visualized using R (3.4.4). Page caching was activated for resource measurements by copying all accessed data to /dev/null before actual program execution. Especially for a short running application like GatherVcfs, runtimes will be noticeably longer without page caching.

### Benchmarking of the entire workflow

Again, reference genome and annotation GRCg6a of *Gallus gallus* were used. Paired end Illumina sequencing data (2 × 125 bp) were downloaded from the ENA, project PRJEB12944, run accessions ERR1303580, ERR1303581, ERR1303584, ERR1303585, ERR1303586 and ERR1303587. Average coverage of these files ranged between 24 and 28. Five intervals were specified in the config.yaml file for parallelization of the HaplotypeCaller (GatkHCintervals). Thereby the reference genome was actually split into six discrete intervals. This is intended behavior of the splitting algorithm, which only splits the reference genome between individual sequences, while trying to limit the maximum size of the created intervals (see createIntervalLists.py for implementation). To measure resource usage of the entire workflow, the commands mpstat 30 and sar -r ALL 30 from the sysstat application suit (12.2.0) were employed. Recorded measurements were plotted using R (3.4.4).

## Availability and requirements

**Table:** 

Project name:	OVarFlow
Project home page:	
- GitLab:	https://gitlab.com/computational-biology/ovarflow
- Read the Docs:	https://ovarflow.readthedocs.io/en/latest/
- Docker hub:	https://hub.docker.com/r/ovarflow/release/tags
- Zenodo (Singularity):	https://zenodo.org/record/4746639
Operating system(s):	tested and working on Linux
	container images might also work on macOS and Windows
Programming language:	Python & Snakemake; Bash
Other requirements:	Conda/Bioconda or Docker/Singularity
License: Code:	GNU General Public License version 3 (GPLv3)
Documentation:	Creative Commons license CC-BY SA 3.0
Any restrictions to use by non-academics:	none

## Data Availability

All sequencing data analyzed in this study are available in the European Nucleotide Archive (https://www.ebi.ac.uk/ena/browser/home) under the following run accession codes: ERR2505843, ERR3525251, ERR3525252, ERR3525253, ERR3525254, ERR3525255, ERR3525256, ERR3525257, ERR3525258, ERR4326765, ERR4326766, ERR4326767, ERR4326768, ERR4326769, ERR4326770, ERR4326771, ERR4326772, ERR4326773, ERR4326774, SRR2131198, SRR2131199, SRR2131201, ERR5079491, ERR5079492, ERR5079493, SRR5079496, SRR3041137, ERR1303580, ERR1303581, ERR1303584, ERR1303585, ERR1303586 and ERR1303587. Chicken genome sequence data (fasta format) and associated annotations (general feature format) were obtained from the RefSeq for the two assemblies Gallus_gallus-5.0 (https://ftp.ncbi.nlm.nih.gov/genomes/all/GCF/000/002/315/GCF_000002315.4_Gallus_gallus-5.0/) and GRCg6a (https://ftp.ncbi.nlm.nih.gov/genomes/all/GCF/000/002/315/GCF_000002315.6_GRCg6a/). Usage of the respective data is outlined within the methods section.

## Abbreviations

Adw: Autosomal dwarfism
AVX: Advanced vector extensions
BQSR: Base quality score recalibration
bwa: Burrows–Wheeler aligner
CPU: Central processing unit
csv: Colon separated values
ENA: European Nucleotide Archive
GATK: Genome Analysis Toolkit
Gb: Gigabytes
GC: Garbage collection
GWAS: Genome-wide association study
indel: Insertion-deletion
JVM: Java virtual machine
NGS: Next generation sequencing
RSS: Resident set size
SGS: Second generation sequencing
SNP: Single nucleotide polymorphism
VCF: Variant call format
WES: Whole exome sequencing
WGS: Whole genome sequencing
